# Effects of Temperature and Time on the Denaturation of Transforming Growth Factor Beta-1 and Cytokines from Bovine Platelet-Rich Gel Supernatants

**DOI:** 10.3390/gels10090583

**Published:** 2024-09-11

**Authors:** Jorge U. Carmona, Catalina López

**Affiliations:** 1 Grupo de Investigación Terapia Regenerativa, Departamento de Salud Animal, Universidad de Caldas, Manizales 170004, Colombia; 2 Grupo de Investigación Patología Clínica Veterinaria, Departamento de Salud Animal, Universidad de Caldas, Manizales 170004, Colombia; catalina.lopez@ucaldas.edu.co

**Keywords:** platelet-rich plasma, platelet-rich gel, bovine, temperature, temporal mediator stability, growth factors, cytokines

## Abstract

There is a lack of information about transforming growth factor beta-1 (TGF-β_1_) and cytokines contained in pure platelet-rich plasma (P-PRP) and release from pure-platelet-rich gel supernatants (P-PRGS) might be affected by the temperature and time factors; P-PRP from 6 heifers was activated with calcium gluconate. Thereafter, P-PRG and their supernatants (P-PRGS) were maintained at −80, −20, 4, 21, and 37 °C and collected at 3, 6, 12, 24, 48, 96, 144, 192, 240, and 280 h for subsequent determination of TGF-β_1_, tumor necrosis factor alfa (TNF-α), interleukin (IL)-2, and IL-6; TGF-β_1_ concentrations were significantly (*p* < 0.05) higher in PRGS maintained at 21 and 37 °C when compared to PRGS maintained at 4, −20, and −80 °C; PRGS TNF-α concentrations were not influenced by temperature and time factors. However, PRGS maintained at 4 °C showed significantly (*p* < 0.05) higher concentrations when compared to PRGS maintained at −20, and −80 °C at 144, and 192 h. IL-6 concentrations were significantly (*p* < 0.05) higher in PRGS stored at −20, and −80 over the first 48 h and at 10 days when compared to PRGS stored at 4, 21, and 37 °C. These results could suggest that P-PRP/P-PRGS could be maintained and well preserved for at least 12 days at room temperature for clinical use in bovine therapeutic massive protocols.

## 1. Introduction

Platelet-rich plasma (PRP), a hemocomponent obtained primarily by centrifugation from anticoagulated whole blood, is a biological product with varying concentrations of “living platelets and leukocytes” that can be activated exogenously or endogenously for its clinical use [1]. This activation process induces the polymerization of PRP into a platelet-rich gel (PRG), which acts in two ways: (1) by generating a biological low-density fibrin gel scaffold that allows cell migration, cell anchoring, and cell differentiation, among others, and (2) by synchronous release of mediators from platelets, leukocytes, and other cells (i.e., resident cells and stem cells) over time [2]. These biological properties make PRP/PRG a versatile bioproduct for treating wounds, infections, fractures, muscle tears, and chronic musculoskeletal disorders in various areas of human and veterinary medicine [3,4,5,6,7,8,9].

There are several taxonomic systems to classify the different types of PRP used in experimental and clinical settings. However, one of the simplest and most widely used PRP classifications considers the concentration of leukocytes in PRP to divide these hemocomponents into two groups: leukocyte- and platelet-rich plasmas (L-PRPs) and pure PRPs (P-PRPs) [10,11]. In general, L-PRPs include those hemocomponents with detectable to high leukocyte concentrations and usually high platelet concentrations compared to the baseline concentrations of these cytoplasmic fragments in whole blood. Conversely, P-PRPs have low to negligible leukocyte concentrations and low to moderate platelet concentrations compared to whole blood [10,11].

Currently, there is interest in the use of PRP for the treatment of reproductive disorders [8,12,13,14] and clinical [15] and subclinical mastitis [16,17] in dairy cows. The use of PRP in bovine practice could be related to the treatment of individual patients where an autologous, small, and fresh volume of PRP may be indicated for the clinical management of a specific condition such as a wound, joint pathology, or subsolar abscess, among others. However, the therapeutic management with PRP of herd-generalized pathologies, such as subclinical mastitis, could require large volumes of allogeneic PRP, which in some cases may not be fresh at the moment of its use.

Therefore, it is of paramount importance to know how time and temperature might affect the stability of growth factors (GFs) and cytokines released from PRG supernatants (PRGS) over a long period. This knowledge could be useful in determining the minimum shelf life of allogeneic bovine PRP, bearing in mind that it is a living hemocomponent, unlike platelet lysates, which are hemocomponents with high concentrations of growth factors and cytokines but devoid of cells and have been frozen for preservation [18].

In line with this, we conducted an in vitro study to know how the time and temperature factors could affect the stability of transforming growth factor beta 1 (TGF-β_1_) and pro-inflammatory cytokines (tumor necrosis factor-alpha (TNF-α), interleukin-2 (IL-2), and IL-6 over a 12-day (288 h) period in allogeneic P-PRP produced for herd massive treatments, such as a program of subclinical mastitis therapeutic management [16,17] or for reproductive procedures [8].

## 2. Results and Discussion

### 2.1. Platelet and Leukocyte Concentrations in Whole Blood, P-PRP, and Plasma

**Figure 1:**
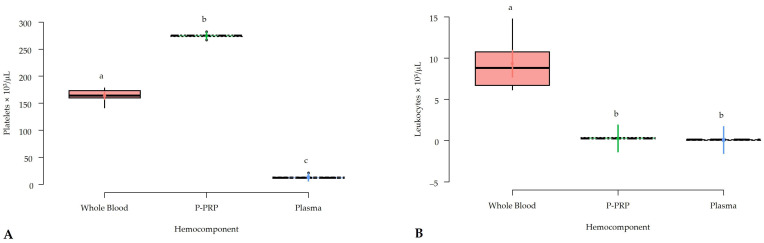
Box plots showing the means and their 95% confidence intervals (95% CIs) for (**A**) platelet and; (**B**) leukocyte counts in different hemocomponents. a–c = different lowercase letters denote significant differences (*p* < 0.001) for the variables evaluated by the Tukey test between hemocomponents. P-PRP = pure platelet-rich plasma.

Platelet and leukocyte counts were significantly (*p* < 0.001) different between whole blood, P-PRP, and plasma, with the highest platelet counts in P-PRP followed by whole blood and plasma (Figure 1A), whereas leukocyte counts were highest in whole blood followed by P-PRP and plasma (Figure 1B). Platelet enrichment in P-PRP compared to whole blood was 1.67-fold, while leukocyte depletion in P-PRP compared to whole blood was 13.9-fold.

Both platelet and leukocyte concentrations obtained in this P-PRP are similar to the obtained for these blood constituents in previous studies [16,17,18], which reflects the reproducibility of the centrifugation protocol to obtain this hemocomponent massively.

### 2.2. Effects of Temperature and Time on TGF-β_1_ Concentrations in Pure Platelet-Rich Gel Supernatants (P-PRGSs)

**Figure 2:**
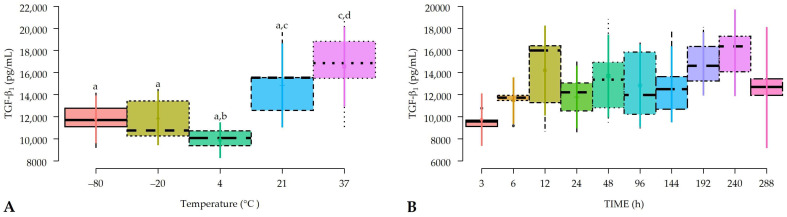
Box plots showing the means and their 95% CIs for (**A**) transforming growth factor beta 1 (TGF-β_1_) concentrations according to the temperature and; (**B**) the time factors. a–d = different lowercase letters denote significant differences (*p* < 0.05) for the variables evaluated by the Tukey test between hemocomponents.

TGF-β_1_ concentrations were significantly affected by the temperature factor but not by the time factor or the interaction between these two factors (Table 1). Regarding the temperature factor, PRGS incubated at 37 °C showed significantly higher concentrations of this growth factor compared to PRGS maintained at −80, −20, and 4 °C. In addition, PRGSs maintained at 21 °C showed significantly higher TGF-β_1_ concentrations than those maintained at 4 °C. Of note, PRGSs maintained at −80, −20, and 4 °C and PRGSs maintained at 21 and 37 °C were similar (Figure 2A).

**Figure 3:**
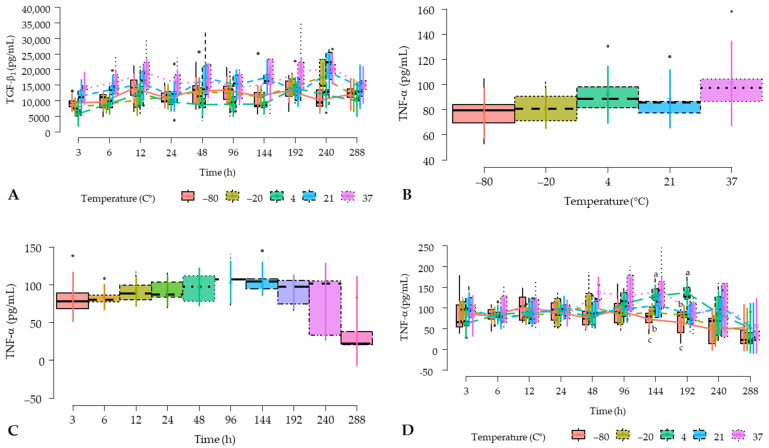
Box plots showing the means and their 95% CIs for (**A**) transforming growth factor beta 1 (TGF-β_1_) concentrations according to temperature and time factor interaction; (**B**) tumor necrosis factor alfa (TNF-α) concentrations according to the temperature factor; (**C**) TNF-α concentrations according to the time factor; and (**D**) TNF-α concentrations according to temperature and time factor interaction. a–c = different lowercase letters denote significant differences (*p* < 0.05) for the variables evaluated by the Tukey test between hemocomponents.

Although non-significant differences were observed for the time factor, the mean concentrations of TGF-β_1_ over time showed a tendency to increase from 3 to 240 h (10 d) and to decrease at 288 h (12 d) (Figure 2B). On the other hand, Figure 3A shows the concentrations for this growth factor considering the interaction between temperature and time factors.

TGF-β_1_ is a pleiotropic and ubiquitous polypeptide that is extremely well-conserved among mammals [19]. This mediator is mainly stored and released from platelet-alpha granules; therefore, its concentrations are measured in PRGS as an indicator of GF enrichment in PRP products [20]. In general, TGF-β_1_ is a homodimeric anti-inflammatory protein with multiple physiological actions, including cell proliferation, cell differentiation, extracellular matrix deposition, and even apoptosis [19,21]. We selected this GF for our study because although it is first massively released from platelets in PRP or PRP-related products, a second wave of this GF is produced by live leukocytes contained in PRG [18,22]. Therefore, this GF may be a good indicator of cell (leukocyte) activity and viability in the PRGs of the present study.

Our results are novel and complement a previous study we conducted on the stability of growth factors and cytokines on bovine PRGS [18]. In the present study, the temperature factor significantly influenced the concentration of this GF, which was highest in PRGS maintained at 37 and 21 °C when compared to PRGS maintained at −80, −20, and 4 °C. This finding implies that TGF-β_1_ remains more stable and in higher concentrations over time in PRGS maintained at 37 °C at least for 12 days. It is important to mention that in a previous study [18], we observed a similar finding in bovine PRGS until 96 h. On the other hand, a similar finding has been described for TGF-β_1_ concentrations obtained in two hemocomponents of equine origin over 8 days [23]. On the other hand, the results of the present study may indicate that TGF-β_1_ concentrations in PRGS were not affected, at least, during the established time for this experiment, although we observed a trend peak of this growth factor at day-10 with a slight decline at day-12.

### 2.3. Effects of Temperature and Time on Pro-Inflammatory Cytokine Concentrations in Pure Platelet-Rich Gel Supernatants (P-PRGS)

TNF-α concentrations were not influenced by the temperature and time factors; however, the interaction between these fixed factors significantly influenced the PRGSs concentrations of this pro-inflammatory cytokine (Table 2).

**Figure 4:**
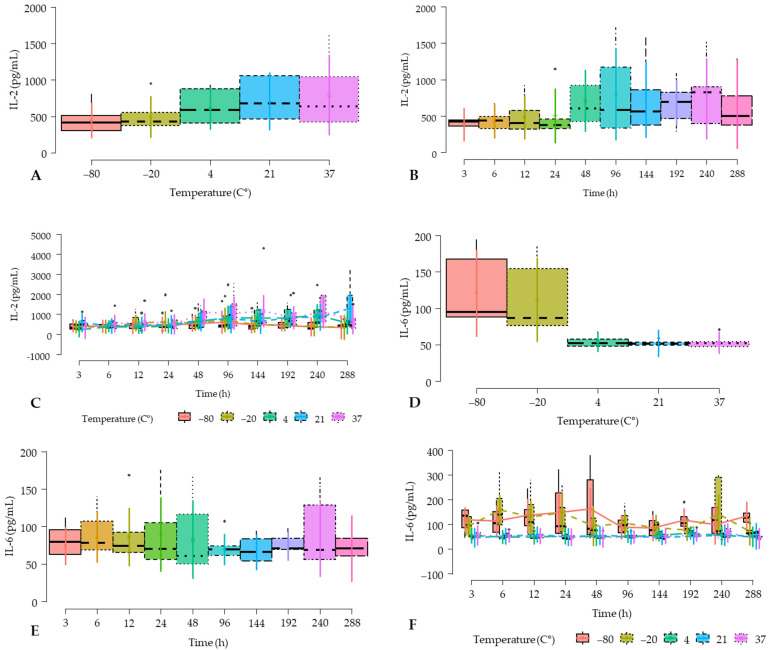
Box plots showing the means and their 95% CIs for (**A**) IL-2 (IL-2) concentrations according to the temperature factor; (**B**) IL-2 concentrations according to the time factor; (**C**) IL-2 concentrations according to temperature and time factor interaction; (**D**) IL-6 concentrations according to temperature factor; (**E**) IL-6 concentrations according to the time factor. (**F**) IL-2 concentrations according to temperature and time factor interaction. Note: For specific statistically significant differences in IL-6 concentrations according to the interaction of temperature and time factors, see Supplementary Table S1.

Figure 3B shows the TNF-α concentrations in P-PRGSs for temperature factor, while Figure 3C shows the concentrations for this cytokine by time factor, and Figure 3D shows the concentrations for this pro-inflammatory cytokine according to the interaction between temperature and time factors. Of note, TNF-α concentrations tend to be higher in PRGSs maintained at 37 °C, while the concentrations for this cytokine tend to increase over the first 10 days to decline in a similar pattern as TGF-β_1_. Regarding the temperature and time interaction, TNF-α concentrations were significantly higher in PRGSs maintained at 4 °C at 144 and 192 h when compared to PRGSs frozen at −80 and −20 °C at the same time points (Figure 4B).

TNF-α is a regulatory cytokine with pro-inflammatory and catabolic effects. Thus, its complete blockade produces death [24,25,26]. Accordingly, the TNF-α response may be associated with the pathogenesis of inflammatory diseases or could be upregulated by bacterial infection. Furthermore, this mediator is a potent inducer of cell death [27,28]. We selected this cytokine for our study because it may indicate the viability of leukocytes in PRGs and how its concentrations may be affected by the temperature factor. Accordingly, in our study, TNF-α concentrations obtained in PRGS were not affected by the temperature and time factors; although PRGS incubated at 37 °C trended to have higher concentrations of this cytokine when compared to the other PRGS maintained at inferior temperatures. This finding could indicate an increased metabolic activity of leukocytes at 37 °C. However, the concentrations for this cytokine in all PRGS remained in the ranges previously described (20–145 pg/mL) for the plasma of healthy bovines [18].

In our study, the time factor did not affect TNF-α PRGS concentrations; however, a trend was observed where peak concentrations of this mediator were observed at 8 days for a subsequent decline. This finding may contradict the results of an equine platelet-rich fibrin study, in which concentrations of this cytokine increased for up to 11 days. [22]. On the other hand, we observed a significant increase in the TNF-α concentrations of PRGS maintained at 4 °C at 6 and 8 days compared to PRGS frozen at −80 and −20 °C for a subsequent decline on the rest of the time points evaluated. Therefore, it is possible that low (cool) temperatures may induce leukocyte stimulation and increased release of inflammatory cytokines. This fact may be an interesting finding that may indicate that a cool environment is not ideal for PRP storage and preservation. In addition, it is important to consider that in a previous bovine P-PRP study [18], both the negative control (plasma) and the positive controls (chemically induced platelet lysate and temperature-induced platelet lysate) presented mean TNF-α concentrations ranging between (81.00 and 99.80 pg/mL) at 0 h, which could indicate that the initial main source of this cytokine is plasma and possibly at that moment the leukocytes present in both positive controls were not stimulated to produce this cytokine [18].

IL-2 concentrations were not influenced in the model by the fixed effects of the temperature, and time factors and their interaction (Table 3). In general, IL-2 concentrations tend to be higher in P-PRGSs incubated at 37 °C and lower in P-PRGSs frozen at −80 and −20 °C (Figure 4A). Similarly, IL-2 concentrations tend to be lower during the first 48 h and then tend to increase until the end of the experiment (288 h) (Figure 4B). Notably, a similar IL-2 concentration pattern was observed when the interaction of temperature and time factors was considered (Figure 4C).

IL-2 is an important regulatory cytokine required for the growth and maintenance of regulatory T cells. Thus, it is important to prevent tumor cell development and to reduce the inflammatory response in autoimmune diseases such as rheumatoid arthritis [29,30,31]. A recent study in horses showed that this cytokine is upregulated on monocyte-derived macrophages stimulated with platelet lysates [32]. On the other hand, we chose this cytokine in our study because we observed that it was increased in the milk of cows with subclinical mastitis treated with P-PRP [16], and its upregulation in milk somatic cells might be associated with the health of the udder [33].

It should be noted that IL-2 concentrations tended to be higher in PRGS maintained at 21 and 37 °C in comparison to the rest of PRGS maintained at lower temperatures. This fact was more evident (although not significant) when the interaction between temperature and time factors was evaluated, showing the highest concentration of this cytokine in PRGS incubated at 37 °C for 10 days and at 21 °C for 12 days. This fact possibly indicates leukocyte activity in PRGs maintained at 21 and 37 °C.

IL-6 concentrations were not affected by the temperature and time factors; however, the concentrations for this cytokine were significantly affected by the interaction between these fixed factors (Table 4).

IL-6 is a pleiotropic pro-inflammatory polypeptide temporally produced after tissue trauma or infection. This cytokine is essential for the activation of defense mechanisms. However, when it is dysregulated, inflammatory conditions become chronic, negatively affecting immune cells [34]. We selected this cytokine in our study to know if it could be affected by temperature and time factors and if these factors could increase the production of this cytokine in PRGS with therapeutic potential for cows with clinical or subclinical mastitis, considering that higher concentrations of IL-6 in PRGS could be harmful to the treated cows because higher concentrations of this cytokine could exacerbate the inflammatory reaction [35,36].

PRGS frozen at −80 and −20 °C tend to present higher concentrations of IL-6 about the same hemocomponents maintained at 4, 21, and 37 °C (Figure 4D). In addition, the concentrations of this inflammatory cytokine remained constant throughout the different time points of the experiment (Figure 4E). However, when the interaction between temperature and time factors was evaluated, PRGS stored at −80 (at 48, 288, 24, 12, 3, and 192 h) and −20 °C (at 6, 24, 240, and 12 h) had the significantly highest IL-6 concentrations compared to PRGS maintained at 4 (at 192, 144, 3, 96, 240, 24, 12, 288, 48, 24 h), 21 (at 12, 96, 192, 6, 48, 240, 144, 288, 24, 3), and 37 °C (at 240, 192, 6, 144, 96, 3, 12, 24, and 288 h) (Figure 4F).

At this point, it should be noted that due to the multiple statistically significant differences observed between PRGS at different temperatures over time, it is not practical to highlight these differences in Figure 4F. However, Supplementary Table S1 shows the mates (means), standard errors, and 95% confidence intervals of the means for PRGS maintained at different temperatures over specific time points.

In the present study, we observed that the temperature factor interaction affected the production of this cytokine with the highest concentrations for PRGS stored at −80 and −20 °C, especially in PRGS frozen over the first 48 h and at 10 days. This fact also indicates that the frozen storage of these hemocomponents increases the concentration and preservation of this pro-inflammatory cytokine, which, as mentioned, could be harmful to tissues or cells exposed to these hemocomponents.

This study had several limitations, such as the few growth factors and cytokines measured in the study, the time points of the evaluations (only 12 days), and the lack of microbiological analysis to determine the possible bacterial contamination of the PRGS evaluated. Regarding this last aspect, at the macroscopic level, PRGS always showed a clear appearance, which could be a qualitative indication that these substances remained free of microbial contamination.

On the other hand, we arbitrarily selected the most plausible and common temperatures at which PRGS could be stored prior to clinical use, including the “classical” freezing temperatures (−80 and −20 °C), the most common chilling temperature (4 °C), the room temperature (20 °C), and the incubation temperature (37 °C). However, additional temperatures above 37 °C should be evaluated, as this type of biologic could be used in dairy herds located in tropical regions with higher temperatures. Another limitation of our study was that histology or transmission electron microscopy evaluations were not performed on PRG clots. However, we had budgetary restrictions that prevented us from evaluating how the structural architecture of the clots is affected by temperature and time factors.

There are several physical and chemical methods to avoid denaturation of mediators in biological substances such as whole blood or other hemocomponents [37,38]. Among the chemical methods to preserve GFs and cytokines in PRGS, protease inhibitors (i.e.,: phenylmethanesulfonyl fluoride (PMSF), EDTA, or a commercially available protease inhibitor cocktail), reducing agents (i.e.,: DTT or β-mercaptoethanol), and protein stabilizers (i.e.,: glycerol, sucrose, or trehalose) could be used [39,40]. However, the addition of these substances to a P-PRP product for mass treatment of herds may not be recommended, especially if the intention is to treat cows with subclinical mastitis or uterine disease, because these chemicals may contaminate milk for human consumption [41] or induce noxious immunological responses in exposed tissues [42,43].

## 3. Conclusions

Allogeneic P-PRP/P-PRG for massive therapeutic use in dairy cows maintained at 21 and 37 °C for 10 days presented a better TGF-β_1_ and cytokine concentration profile than P-PRP/P-PRG refrigerated at 4 °C or frozen at −80 or −20 °C. P-PRP/P-PRG cooled at 4 °C exhibited the worst TGF-β_1_ concentrations with a possible higher denaturation of this polypeptide, while the hemocomponents frozen at −80 or −20 °C exhibited the highest IL-6 concentrations. These results could suggest that P-PRP/P-PRG could be maintained and well preserved for at least 12 days at room temperature for clinical use in cows with subclinical mastitis. However, further studies are needed to stabilize the denaturation of a broader array of growth factors and cytokines at different temperatures over a longer period, as well as to determine the bacteriological quality of these hemocomponents over time.

## 4. Materials and Methods

This study was approved by the Animal Experimentation Committee of the Universidad de Caldas, Manizales, Colombia (Authorization code 16.06.16.01). The cows included in the study came from one of the farms of the institution and were under the responsibility of the researchers.

### 4.1. Animals

Six clinically healthy *Blanco Orejinegro* heifers with a mean age of 24 months (range: 16–30 months) and a mean weight of 250 (±60 kg) were included. The health status of each heifer was assessed by physical examination, automated complete blood count (CBC), urinalysis, and renal and hepatic clinical chemistry panels. The animals were dewormed and housed overnight and grazed in small paddocks during the day for two weeks before the start of the experiment. They were fed with kikuyu grass, 2 kg of concentrate, fresh water, and mineralized salt ad libitum.

### 4.2. Blood Procurement and P-PRP Processing

Blood collection and P-PRP procurement were performed according to a previous study [18]. Briefly, the heifers were sedated and restrained for the aseptic collection of 450 mL of whole blood into double transfusion bags containing CDPA-1 as an anticoagulant by jugular venipuncture. A 10 mL blood sample was collected from each bag to perform a baseline complete blood cell count (CBC) (Celltac α MEK-6450. Nihon Kohden, Tokyo, Japan). After blood collection, the heifers were closely monitored and kept in a quiet, warm stall until they fully recovered from sedation. They were then returned to the paddock to receive feed and water.

Transfusion bags were centrifuged at 698 g for 6 min at room temperature (21 °C) in a stationary centrifuge (RotoSilenta 630 RS. Hettich, Tuttlingen, Germany). The plasma from each bag was gently separated and packed into the satellite bag (220–240 mL). This plasma was considered P-PRP.

### 4.3. Study Design

P-PRP collected from the satellite bags was placed in 10 mL plastic tubes and activated with a 10% calcium gluconate solution (9.3 mg/mL) (Ropsohn Therapeutics Ltd.a^®^, Bogotá, Colombia) at a 9:1 ratio to induce polymerization of P-PRP to P-PRG and subsequent release of mediators. P-PRP samples were incubated with calcium gluconate for 3 h to induce P-PRG. P-PRG was then maintained at −80, −20, 4, 21, and 37 °C at 6, 12, 24 (1 d), 48 (2 d), 96 (4 d), 144 (6 d), 240 (10 d), and 288 (12 d). PRG supernatants (P-PRGS) produced at each time point were collected individually from each tube after centrifugation at 5000 g for 5 min. In addition, frozen PRGS at −20 and −80 °C were thawed at room temperature at each specific time point, centrifuged, and collected. All P-PRGS produced from tubes in the study were stored at −80 °C for subsequent mediator determination.

### 4.4. Growth Factor and Cytokine Assessment in P-PRG Supernatants

Transforming growth factor beta-1 (TGF-β_1_), tumor necrosis factor-alpha (TNF-α), interleukin-2 (IL-2), and IL-6 concentrations in plasma and PRGS were measured by ELISA in duplicate using development kits from R&D Systems (Minneapolis, MN, USA). TGF-β_1_ was determined using human antibodies (Human TGF-β1 DuoSet, DY240E, Minneapolis, MN, USA) because of the high sequence homology between bovine and human species [44]. Of note, a similar ELISA kit has been used and validated for the same purposes in other bovine PRP studies [16,18,45].

TNF-α (Bovine TNF-alpha DuoSet ELISA, DY2279, Minneapolis, MN, USA), IL-2 (Bovine IL-2 DuoSet ELISA, DY2465, Minneapolis, MN, USA), and IL-6 (Bovine IL-6 DuoSet ELISA, DY8190, Minneapolis, MN, USA), were assayed using species-specific bovine antibodies for these mediators. The standards provided with each ELISA kit were used to construct each standard curve according to the manufacturer’s instructions. Absorbance readings were performed at 450 nm [16,18,45].

### 4.5. Statistical Analysis

Data were analyzed using the free statistical software JASP (JASP (Intel 0.18.3), University of Amsterdam, The Netherlands). Cell data from whole blood, P-PRP, and plasma were compared using a generalized linear mixed model (GLMM) in which the effect of hemocomponent fixed factor was evaluated on platelet and leukocyte concentrations. On the other hand, GLMMs were also used to evaluate the effect of the time (10 levels) and temperature (5 levels) fixed factors on growth factor and cytokine concentration. The interaction between the fixed factors was also included in each model. All models were run using the Gaussian family of distributions with identity as the link function. In addition, cow ID was declared as a random factor. When models showed significant differences, a Tuckey post hoc test was performed to contrast means by fixed factors and their interactions. A *p* < 0.05 was considered statistically significant for all tests performed.

Sample size and power were calculated from the results of a similar study [18]. The number of cows (n:6) used in the study allowed to obtain a statistical power (β) of 0.8 with a significance value (α) of 0.05.

## Figures and Tables

**Table 1:** Generalized linear mixed model (GLMMs) evaluating the effect of the fixed factors and their interactions on TGF-β_1_ concentrations.

Fixed Effect	df	ChiSq	*p*-Value
Temperature	4	9.519	0.049
Time	9	15.278	0.084
Temperature × Time	36	47.680	0.092

df, degrees of freedom; ChiSq, Chi square critical value.

**Table 2:** Generalized linear mixed model (GLMMs) evaluating the effect of the fixed factors and their interactions on TNF-α concentrations.

Fixed Effect	df	ChiSq	*p*-Value
Temperature	4	7.533	0.110
Time	9	15.088	0.089
Temperature × Time	36	97.273	<0.001

Acronyms as in Table 1.

**Table 3:** Generalized linear mixed model (GLMMs) evaluating the effect of the fixed factors and their interactions on IL-2 concentrations.

Fixed Effect	df	ChiSq	*p*-Value
Temperature	4	5.254	0.262
Time	9	5.982	0.742
Temperature × Time	36	46.981	0.104

Acronyms as in Table 1.

**Table 4:** Generalized linear mixed models (GLMMs) evaluating the effect of the fixed factors and their interactions on IL-6 concentrations.

Fixed Effect	df	ChiSq	*p*-Value
Temperature	4	6.820	0.146
Time	9	6.123	0.728
Temperature × Time	36	81.264	<0.001

Acronyms as in Table 1.

## Data Availability

The original contributions presented in the study are included in the article/Supplementary Material, further inquiries can be directed to the corresponding author.

## Supplementary Materials

The following supporting information can be downloaded at: https://www.mdpi.com/article/10.3390/gels10090583/s1, Table S1: interleukin-6 concentrations according to the interaction between time and temperature fixed factors.
