# Supplementary material for: Effects of Temperature and Time on the Denaturation of Transforming Growth Factor Beta-1 and Cytokines from Bovine Platelet-Rich Gel Supernatants
Source: Gels. 2024 Sep 11;10(9):583. doi: 10.3390/gels10090583 (PMC11431824; doi:10.3390/gels10090583)
Supplement: Supplementary file 1 [file gels-10-00583-s001.zip › gels-3201529-supplementary.pdf]

gels

MDPI

Article

Effects of Temperature and Time on the Denaturation of Transforming Growth Factor Beta-1 and Cytokines from Bovine Platelet-Rich Gel Supernatants

Jorge U. Carmona [1,*] and Catalina López [2]

1 Grupo de Investigación Terapia Regenerativa, Departamento de Salud Animal, Universidad de Caldas, Manizales 170004, Colombia
2 Grupo de Investigación Patología Clínica Veterinaria, Departamento de Salud Animal, Universidad de Caldas, Manizales 170004, Colombia; catalina.lopez@ucaldas.edu.co
* Correspondence: carmona@ucaldas.edu.co; Tel.: +57-3136601010

Supplementary

Table S1. Interleukin-6 concentrations according to the interaction between time- and temperature-fixed factors.

				95% CI	
**Time (h)**	**T (°C)**	**Estimate**	**SE**	**Lower**	**Upper**
3	-80	119.23 A	21.58	76.92	161.53
6	-80	114.46	26.85	61.82	167.09
12	-80	137.24 B	28.31	81.75	192.73
24	-80	148.45 C	33.12	83.53	213.37
48	-80	163.13 D	34.38	95.73	230.53
96	-80	89.59	21.68	47.09	132.10
144	-80	85.95	22.88	41.09	130.81
192	-80	117.48 E	21.01	76.30	158.67
240	-80	99.58	32.97	34.95	164.22
288	-80	133.99 F	22.27	90.34	177.64
3	-20	94.865	21.01	53.67	136.05
6	-20	159.74 G	25.90	108.96	210.52
12	-20	131.46 H	28.00	76.58	186.34
24	-20	148.35 I	33.03	83.61	213.09
48	-20	94.18	33.31	28.88	159.47
96	-20	105.76	20.72	65.14	146.39
144	-20	87.50	22.03	44.31	130.69
192	-20	78.10	19.84	39.20	117.01
240	-20	143.54 J	31.86	81.09	205.98
288	-20	72.09	22.28	28.42	115.76
3	4	55.37	14.36	27.22	83.52
6	4	49.32	13.40	23.04	75.59
12	4	49.21	14.54	20.71	77.71
24	4	53.57	16.33	21.55	85.59
48	4	50.99	17.18	17.31	84.67
96	4	50.81	12.53	26.24	75.38
144	4	56.22	12.37	31.96	80.49
192	4	65.40	12.65	40.60	90.19
240	4	55.82	16.65	23.17	88.47
288	4	50.30	15.18	20.54	80.07

Time (h)	T (°C)	Estimate	SE	95% CI	
				Lower	Upper
3	21	46.52	15.97	15.22	77.82
6	21	52.42	14.91	23.19	81.65
12	21	58.88	14.50	30.44	87.31
24	21	49.06	16.05	17.59	80.53
48	21	56.01	16.82	23.04	88.98
96	21	50.84	13.74	23.89	77.79
144	21	47.50	13.81	20.43	74.57
192	21	51.89	14.53	23.40	80.37
240	21	55.95	16.96	22.70	89.20
288	21	52.73	17.69	18.02	87.442
3	37	55.99	16.36	23.93	88.06
6	37	53.49	12.85	28.30	78.68
12	37	53.54	15.26	23.62	83.45
24	37	48.62	16.48	16.32	80.93
48	37	48.10	17.86	13.09	83.11
96	37	49.55	12.90	24.25	74.85
144	37	52.25	13.60	25.59	78.92
192	37	55.77	12.49	31.27	80.26
240	37	65.43	16.48	33.12	97.73
288	37	42.78	15.77	11.87	73.69

A, significantly different (SD) ($p < 0.05$) from PRGS maintained at 4 (6, 12, and 288 h), 21 (at 288 h), and 37 °C (at 288 h). B, SD ($p < 0.05$) from PRGS maintained at 4 (at 192, 144, 6, 12, 288, 48), 21 (at 96, 192, 6, 48 h), and 37 °C (at 240, 192, 6, 96, 12, 24, and 288 h). C, SD ($p < 0.05$) from PRGS maintained at 4 (at 192, 144, 6, 12, 288, 48), 21 (at 96, 192, 6, 48, 288 h) and 37 °C (at 240, 48, 6, 96, 12, 24, 48, and 288 h). D, SD ($p < 0.05$) from PRGS maintained at 4, 21, and 37 °C at all time points. E, SD ($p < 0.05$) from PRGS maintained at 4, (at 48 h), 21 (at 288 h), and 37 °C (at 6, and 288 h). F, SD ($p < 0.05$) from PRGS maintained at 4 (at 192,144, 3, 6, 24, 12, 288, and 48 h), 21 (at all time points), and 37 °C (at all time points). G, SD ($p < 0.05$) from PRGS maintained at 4, 21, and 37 °C at all time points. H, SD ($p < 0.05$) from PRGS maintained at 4, (at 12, and 48 h), 21 (at 6 h), and 37 °C (at 288 h). I, SD ($p < 0.05$) from PRGS maintained at 4 (at 192,144, 3, 6, 24, 12, 288, and 48 h), 21 (at all time points), and 37 °C (at all time points). J, SD ($p < 0.05$) from PRGS maintained at 4 (at 192, 144, 6, 12, 288), 21 (at 96, 192, 6, 48, 288 h) and 37 °C (at 240, 192, 144, 6, 96, 12, 24, 48, and 288 h).
